# Graph genomes article collection

**DOI:** 10.1186/s13059-019-1636-0

**Published:** 2019-02-01

**Authors:** Michael C. Schatz, Andrew Cosgrove

**Affiliations:** Genome Biology, London, UK

*Genome Biology* is pleased to call for submissions to an article collection of methods related to the analysis of genomic data in the context of graph-based genome representations.

In 2015, *Genome Biology* published a comment article calling for the community to adopt graph-based reference genomes and develop methods and protocols to accommodate these graphs [1]. This was shortly after the release of the “Genome Reference Consortium Human Genome (build 38)” (GRCh38) in December 2013, which took a graph-based approach.

The initial human reference genome was a single, haploid sequence. Although having any kind of reference was a great help for many applications, and furthered our understanding of human genetics immeasurably, this “one size fits no one” approach was less than ideal. Since the reference was assembled from the DNA of several subjects, it did not match any one person. In addition, as more and more genomes were sequenced, from different global populations, it became clear that many of the sequences present in the reference were actually quite rare across the species.

Additionally, recent improvements in sequencing technology, and in particular the development of long-read sequencers, have made it easier to discover major structural variations. These duplications and rearrangements were often missing from the linear reference genome and are not well represented in that format.

To get around these limitations in the linear reference, in February 2009, GRCh37 introduced, in a very limited way, a graph-based approach, where certain loci with common variants were represented by alternative sequences. This was extended in GRCh38, in which 3.6 Mbp of sequence was added and spread over 178 loci with at least 1 alternative sequence. This novel sequence contained over 150 genes.

The authors of our 2015 comment noted that, although this graph provided a more complete representation of the reference genome, it added complications. New tools were needed, as applications for things such as read mapping or variant calling could fail when presented with two alternative sequences at the same locus. In addition, new standards were needed for identifying loci, as a single, linear coordinate system no longer worked. Since then, progress on such new methods has been slow, although they have recently started to appear. It has been clear from conferences that the *Genome Biology* editors have attended this year that we can expect more of these methods to be available in the near future, and the NIH has recently announced funding for developing such methods [2].

Given the recent progress, we think that it is the right time for *Genome Biology* to launch an article collection on this topic. Authors are invited to submit any methods relevant to the construction and analysis of graph-based genome representations, or related to the new standards or data structure required for the graphs. Articles will not be delayed by being held for publication together, but will be published as they are ready, once they have been through peer review.

The article collection will be guest-edited by Michael Schatz of Johns Hopkins University. Professor Schatz co-authored the 2015 editorial and is well known for his work developing computational tools for genome assembly and structural variation analysis using new sequencing technologies. He will provide advice and guidance for the special issue working closely with the *Genome Biology* editorial team and reviewers.

For further information, or if you would like to submit a presubmission inquiry, please email editorial@genomebiology.com.
